# Innovative financing of the sustainable development goals in the countries of the Western Balkans

**DOI:** 10.1186/s13705-022-00340-w

**Published:** 2022-02-22

**Authors:** Igor Lukšić, Bojana Bošković, Aleksandra Novikova, Rastislav Vrbensky

**Affiliations:** 1 University of Donja Gorica, Oktoih 1, Podgorica, Montenegro; 2 Institute for Climate Protection, Energy and Mobility (IKEM), Magazinstr. 15-16, 10179 Berlin, Germany; 3 AvantGarde Energy (AGE), Podjavorinskej 7, 811 03 Bratislava, Slovakia; 4 Masaryk University, Brno, Czech Republic; 5 Bratislava, Slovakia

**Keywords:** Sustainable/environmental social governance [ESG] finance, Green bonds, Debt swaps, Climate finance, Public debt

## Abstract

**Background:**

This paper is related to the current stage of development in the Western Balkans. Despite becoming growing instruments to finance sustainable green development, debt swaps and social or sustainability bonds are relative novelties in this region. At the same time, the development needs are huge, especially in the light of the COVID-19 aftermath.

**Results:**

The review of both historic financial instruments, such as the debt for nature swaps, and more recent ones, such as sustainability bonds in its variations, highlight the potential for use in developing countries. The relatively recent case from Montenegro and the recent issuance of the green bond in Serbia showcase the possibilities. The focus of this paper is an analysis of the public debt position of Western Balkan countries. The growing level of public debt over the past decade points to a lack of adequate interventions and a relatively imminent need for fiscal consolidation. The research suggests that environmental, social, governance/sustainability-linked bonds and debt-for-climate swap investments as innovative financial instruments that hold promise in leveraging additional finance to support the sustainability goals of the six countries of the Western Balkans. This influx of capital would be particularly advantageous, given their needs relative to EU accession and their economic and structural challenges. The recommendations for policymakers are derived based on the history and features of green bonds as well as debt-for-nature swaps and their diverse underlying mechanisms which are adaptable to the respective countries.

**Conclusions:**

The related countries would benefit from exploring more innovative approaches to finance sustainable societies. In close cooperation with the EU and taking the European Green Deal into consideration, it is recommended that the six countries of the Western Balkans design financing mechanisms that will bring increased transparency to the different policies and more accountability for their implementation. Applying the recommended modality may help keep the problem of the public debt at bay, while additional funds may support implementation of structural reforms.

## Background

The adoption of the seventeen sustainable development goals (SDGs) in 2015 by the UN General Assembly marked the beginning of a new era for global policy coordination. Following the Millennium Development Goals (MDGs), adopted in 2000, the Sustainable Development Goals, or UN 2030 Agenda, became the anchor agenda for all stakeholders.

As illustrated in a number of studies, the first global policy agenda was only partially met. For example, only 20% and 7% of low- and middle-income countries were on track to meet child and maternal mortality fast-track targets [1]. Despite the fact that MDGs have not fully been met and mostly focused on less developed countries, it was decided that the time was right to move to the next global policy level. The United Nations (UN), individual countries, corporations, multilateral financial institutions and everyday people across the globe and most affected by the success or failure of the goals, came together to set the priority and direction for the SDGs.

Although academic literature on SDG financing and its challenges appears substantially limited [2], the implementation of the UN 2030 Agenda is evidently costly and according to a UN study is estimated to cost between USD 3.3–4.5 trillion per year to fund different projects, development programs and various initiatives which help countries achieve these ambitious goals [3]. According to the same study, developing countries face an average annual funding gap of USD 2.5 trillion. It is clear that government-driven or multilateral aid institutions-led support needs to be complemented by the private sector and the abundant funds that are available on the markets. The task for policymakers and private sector investors is, therefore, to coordinate and look for more innovative approaches. As far as the European region is concerned, the adoption of the European Green Deal is a game changer which sets the stage by introducing clear goals and investment needs to turn the economy around by 2050 [4].

Innovative instruments are needed to scale up international finance for sustainability purposes, but only limited options are available for developing and transitional economies [5]. For instance, the largest share of adaptation finance was provided through grants (77%), while concessional loans (17%) and blended grant/loan and other instruments (6%) only played a subordinate role [6]. Mitigation finance was provided mostly through loans and blended finance instruments [6]. From one perspective, many bi- and multilateral donors report a challenge of disbursing their funds as they fail to identify fundable projects. On the other hand, many developing countries report difficulties in accessing available resources due to lack of capacity and the inability to fulfil specific requirements established by donors or financing institutions [6]. Other experts [7] recommend implementing a set of simple financial mechanisms to address the SDG financing gap quickly and at a transformative scale. These mechanisms include, inter alia, the issuing of sovereign green bonds, SDG lending certificates and rediscounting policies. High external debt burdens further hamper many developing countries in accessing finance and setting their economies on a sustainable path.

However, expectations that blended finance can bridge the SDG financing gap and mobilize private investment in SDGs at scale are currently unrealistic. Each $1 of MDB and DFI invested mobilizes, on average, $0.75 of private finance for developing countries. Blended finance may tip the balance, but it will likely not be effective if the economic fundamentals are not in place. Therefore, the push for blended finance should not divert the attention from the need for grants to boost local investment environments. Donors, therefore, need to think carefully about the allocation of ODA and the risks and trade-offs of investing ODA in blended finance [8]. There is also a need to incorporate many synergies and trade-offs, including around financing, inherent in the goals into systems models. This will help ensure that addressing and financing one goal does not inadvertently impact the ability to achieve others. Though some models are starting to incorporate climate impacts and land and water use in their analysis, few models go beyond this scope to address the many different interactions envisioned by the SDGs [9].

In the meantime, the countries of the Western Balkans Six [Albania, Bosnia and Herzegovina, Kosovo, Montenegro, North Macedonia, and Serbia] struggle to juggle all the policymaking tools to achieve sustainable economic development. In parallel, they are expected to meet benchmarks in a number of complex fields, while achieving good grades related mostly to the rule of law, quality public administration and economic governance to join the EU.

One of the aspects that will be under particular scrutiny is the debt portfolio of the countries in question and the possibilities to improve the current situation, given the sustainable development requirements. This is of particular importance as it is important to address potential that lies ahead in using more innovative means of financing the development policy needs.

Given the fact that early green bonds’ attempts are more than a decade old [10], this new mechanism of issuing green, social or sustainability bonds [including the sustainability linked variation], has been gaining traction in recent years globally. However, it still represents a novelty in the region of the Western Balkans, where countries have mostly been oriented to the classic bond market for the budget needs and international financial intermediary’s [IFI] project financing for other purposes. The reliance on the IFI’s, at least those based in Europe and the World Bank group, has spurred the implementation of certain standards which have steered countries towards indirectly complying with some of the principles of sustainable development.

Debt-for-nature swaps [and swaps for other sustainability purposes] offer another solution to avert both debt and sustainability challenges by providing debt relief alongside mobilizing new finance for achieving sustainability goals. While respective designs vary, all debt swaps share the same underlying mechanism: public debt of a developing country is cancelled in exchange for investments in projects linked to nature protection within the debtor country. There have only been a few debt-for-nature swaps in countries of other world regions so far, and the investigative team recorded one debt swap in Montenegro, but could not identify a recent surge in scholarly attention.

The aim of this paper is to assess new sustainability-linked bonds and debt-for-climate swaps as innovative financial instruments, promising to leverage additional finance into sustainability goals in the Western Balkan Six. As a first step, an online search was conducted for published material on sustainability bonds and debt swaps by researchers, international institutions and think tanks. This also entailed the collection of reports, news articles, and web pages. All collected data was then reviewed and analysed from the perspective of drawing useful recommendations for the focus countries. Second, we analysed relevant data related to the debt situation in the countries and drew conclusions on the usability of instruments based on our experience in policymaking in the region. The subsequent section outlines recommendations for policymakers in designing future green bonds and debt-for-nature swaps and applies these to national circumstances in the Western Balkan Six. This study ends with conclusions highlighting key messages.

### Green, social and sustainability bonds

#### History of green, social, and sustainability bonds

All the new forms of the bonds are voluntary. However, the growing trend is obvious. Investors tend to identify the benefits of a particular intervention based on their interest to approach markets in different ways, the new instrument raises the country or corporation’s visibility or the innovation opens new financing channels as more and more investments funds are committed to keep share of their investments in the green, social or sustainability bonds [11]—or as they are more and more referred, ESG^1^ instruments.

One of the consequences of COVID-19, related to this new bond market, was the slow-down of the green bonds against the growth of the social and sustainability bonds. The social bonds hit a record with an increase of 170% since the beginning of 2020. However, despite rising corporate interest, the rapid increase in social bond issuances as a response to the recent pandemic has been mainly led by IFIs and primarily multilateral development banks (MDBs) [10]. In parallel, there is an expectation that there will be a need to restructure sovereign debt in a number of countries for which a more innovative approach may be needed [12].

The Green Bonds Principles just like the Social Bonds Principles or the Sustainability Bonds Principles are designed to promote the transparency and integrity needed to increase capital allocation to the projects that highlight either the green or social element [or the mix]. These investments are thus linked to the sustainability policy, as explained by the International Capital Markets Association’s briefs [13, 14]. To muster more resources needed for the SDG financing, the best avenue to take is to increase transparency of the financing goals and the policy makers’ directions in the mid- to long-run.

Launched in 2007, with the European Investment Bank and the World Bank, issuance of the green market bond has grown significantly. The wider bond market started to react after the first USD 1 billion green bond sold within an hour of issuance by IFC in March 2013, while in November of the same year, corporate green bonds started to see the light.^2^ The key year was 2014 when USD 37 billion worth bonds were issued, while the new record was set in 2019 when issuance reached almost USD 259 billion. The cumulative issuance since 2007 stands at USD 754 billion across 5931 deals and 927 issuers, while so-called certified climate bonds reached the USD 100 billion milestone [15].

Normally, the projects funded are related to the climate change mitigation, climate change adaptation, natural resource conservation, biodiversity conservation, and pollution prevention and control, and are focused, for example, on energy or emissions reduction projects, sustainable agriculture, and green buildings.

On the other hand, social bonds are intended to meet various social needs. Their role is to provide capital for projects that contribute to socioeconomic advancement and empowerment—such as affordable housing and infrastructure, access to essential services, employment generation, and food security [16].^3^ They are similar in structure to green bonds, a particularly popular form of “use of proceeds” bonds [10]. Logically, the target populations include people living under the poverty line, undereducated communities, marginalized groups and so on.

All of these new instruments require vigilant reporting by external parties who monitor and certify accomplishments which adds to their credibility and makes them potentially preferred mechanisms in addressing the mid- to long-term needs to meet SDG benchmarks and help decarbonize the economy while making best use of private–public partnerships. However, the magnitude of the need is very large. Communicating the European Green Deal in December 2019, the Commission estimated the need for an additional EUR 260 billion per year or about 1.5% of 2018 GDP to reach energy and climate 2030 goals [4]. It is obvious that the new financing mechanism needs to be given a boost.

The current total value of outstanding green, social and sustainability bonds is USD 1503 billion [17]. However, as the IFC team points out in their notes; green, social and sustainability bonds still only make up a fraction of the overall bond market. Compared to green bonds, the social bond market is still in its nascent stage. However, issuances have skyrocketed since the outbreak of COVID-19 in early 2020, as social bonds have become of increasing interest to investors looking to achieve positive social outcomes together with a financial return [10].

There is still much progress to be made when considering the regulatory framework, especially now that sustainable finance is becoming a part of mainstream discourse. The green transition in various sectors can be significantly supported by some of these sustainability or performance linked mechanisms. Therefore, it is expected that the EU will further work in this field by creating an EU Green Bond Standard [18], including defining EU Taxonomy [19] with reference to the guidelines and disclosures. In the meantime, Climate Bonds Initiative has launched Climate Bonds Standard 3.0, aiming to improve the overall business environment [15].

#### Examples of sustainability bonds worldwide

Demand for green bonds is continuously increasing. Until roughly 2012, the green bond market was dominated by multilateral development banks, which already had in place processes for assessing environmental, social and governance [ESG] risks for projects. This has, however, changed over time, with a growing number of green bond issuances by corporations, energy and utility companies and governments and their agencies from around the world. Notable examples include Chinese issuers, who in 2016 comprised about 40% of the overall green bond market, as well as Poland, which in the same year became the first sovereign state to issue a green bond, followed by France, which issued the largest ever and longest dated benchmark green bond, a Euro 7 billion, 22-year benchmark bond. In 2017, while holding the COP23 Presidency, Fiji became the first sovereign emerging market issuer of green bonds when it issued a green bond with a value of USD 50 million [20].

In the context of this paper it is worth mentioning that Serbia issued their first ever green bond in September 2021 becoming the first Western Balkans country to do so. It issued a 1 billion USD worth green bond. The 7-year maturity and 1% annual coupon security is aimed at investments in the rail and subway network, sewerage, water and wastewater processing, flood protection, biodiversity protection, pollution prevention and control, waste management and at providing support for energy efficiency measures and the installation of rooftop solar panels [21].

According to [22], the global green bond market in 2019 grew by 51% to reach $260 billion with loan proceeds primarily used in clean energy, building, and transport sectors mainly in the EU and Asia Pacific, and North America. By far and large, this innovative green finance instrument has not reached the developing countries at the scale and in the sectors required—where capital is most needed. The challenge, therefore, is to expand the issuance of green bonds to developing countries. One of the recommendations made in this respect for the governments in developing economies is to adopt a holistic and comprehensive policy framework that is conducive to the inflow of sustainable investment resources, offers investors protection, and is transparent. It is also critical to clearly and transparently link performance indicators with environment and sustainability. This includes developing sovereign green bonds as well as encouraging private enterprises to issue green bonds to fund climate adaptation and mitigation efforts.

To date, more than 90% of all new green bonds have come from issuers other than multilateral development banks. This is illustrated, for instance, by the ranking of the largest climate bond issuers in 2019. Fannie Mae, the pioneer of issuing Green Mortgage-backed Securities [MBS], remained the largest green bond issuer with 22.9bn USD issuance or 9% of the total. KfW, the German state-owned development bank, was the second largest issuer with a total of 9bn USD worth of green bonds in the market with proceeds used to provide financing or co-financing to renewable energy and green building projects. They were followed by the Dutch State Treasury Agency [DSTA] ranked as the third largest issuer with 6.7bn USD debut green sovereign bond.

Another recent example of this phenomenon was the announcement of global biopharmaceutical company Pfizer about launching a $1 billion USD sustainability bond offering, with the use of proceeds funding R&D and capex for the company’s COVID-19 vaccine. The 10-year bond was priced at 1.75%. The issue marks the second for Pfizer under its Sustainability Bond Framework, following a $1.25 billion USD 2.625% 10-year offering in March of last year. According to the company, proceeds from the offering will be used to finance or refinance research and development expenses related to COVID-19 vaccine research and development, capital expenditures in connection with the manufacturing and distribution of COVID-19 vaccines, and other projects of Pfizer or any of its subsidiaries that have environmental and/or social benefits [23].

#### Incentives for green bonds issuance

Although to date, there has been very little academic work in the theory of the growth of the green bond market in the academic literature [24], some conclusions can already be gleaned. In general, the financial incentives for investing in green bonds are no different than for other asset classes. An investor has financial incentive to invest in a green bond if this bond provides some or all the following benefits: better returns, lower risk, and better diversification benefits than other comparable assets. An issuer has a direct financial incentive to issue a green bond if the green bond reduces their cost of capital and/or improves their access to capital.

Besides incentives related to the economic performance of the investor there are incentives that are not directly related to the financial performance of the green bond. [25] The four types of non-financial business case incentives are: operational, efficiency branding, creating new markets, and reducing risk. Operational efficiency could be enhanced by attracting high quality employees or making an impact on the productivity of employees motivated by sustainability commitments [26]. The branding benefits of engaging in sustainable finance include attracting and retaining customers or charging premium prices for products and services [27].

Creating new markets could entail developing new investment products for customers interested in sustainable investing and/or attracting new classes of customers to existing and new product offerings [28]. There are also incentives not directly related to financial risks such as those associated with reducing reputational risks and risks associated with potential future regulatory framework related to sustainability [29]. In addition, the academic literature identifies additional incentives, associated with broader forces, such as the legitimacy of the organization and institutional-oriented drivers connected to operating at a societal level [30, 31].

#### Features of sustainability-linked bonds

A variation to the above-mentioned bonds is the sustainability-linked bonds [SLBs] or ESG bonds. The proceeds of SLBs are intended for general purposes and due to the variability of the coupon, based on the accomplishment, may be further explored by the Western Balkan countries. During the preparation process objectives are measured through predefined key performance indicators [KPIs] and assessed against predefined sustainability performance targets [SPTs].

The Sustainability-Linked Bond Principles [SLBP] [14], established by ICMA, recommend that issuers publicly communicate their rationale for the selection of their KPIs [i.e., relevance, materiality], the motivation for the SPTs [i.e., ambition level, consistency with overall strategic planning and benchmarking approach], the potential change of bond financial and/or structural characteristics and the trigger events leading to such a change, intended post issuance reporting and independent verification. The target setting exercise should be based on a combination of benchmarking approaches that include the issuers’ own performance, the performance of its peers and reference to the science. In this process, the issuer may seek a second party opinion.

The potential variation of the coupon is the most common example, but it is also possible to consider the variation of other SLB’s financial and/or structural characteristics.

What adds to the transparency and the overall credibility of this mechanism is that issuers should seek independent and external verification [for example limited or reasonable assurance] of their performance level against each SPT for each KPI by a qualified external reviewer with relevant expertise, such as an auditor or an environmental consultant, at least once a year.

### Debt-for-nature swaps

#### History of debt-for-nature swaps

Reference [32] provide a historical review of the debt-for-nature swaps. According to the authors, the design and use of the debt swap instrument dates back to the 1990s with the first instruments taking the form of debt-for-equity swaps. Debt restructuring started almost immediately after World War II and accelerated when the Paris Club^4^ was founded in 1956 [33]. Composed of officials from major creditor countries, the club would initiate the first large-scale debt relief programs in the form of debt-for-equity swaps, and from 1991 onwards allow debtors to convert their public debt into local payments for social or environmental projects [34]. The first debt-for-equity swap took place in Chile in 1985, where commercial debt was cancelled in exchange for creditors receiving shares in publicly owned enterprises [35].

The first debt-for-nature swaps appeared in the 1980s as a response to the worsening environmental conditions in developing countries. In the 1980s many countries particularly in Latin America increased their export volume in response to the debt and economic crisis that caused heightened rates of resource exploitation and deforestation.^5^Soon after, the first debt-for-nature swap was agreed upon by Bolivia and Conservation International, in which the latter bought USD 650,000 of Bolivia’s commercial debt at a redemption price of USD 100,000 from a Swiss bank. This debt was then swapped against a commitment by the Bolivian government to spend USD 260,000 on biodiversity conservation [35].

The volume of debt swaps grew in the 1980s to peak in 1990 and decline through the 1990s. Throughout the 1980s, the secondary market of debt titles grew rapidly and debt swaps were advertised as a standard instrument of debt relief by lending agencies seeking new ways to minimize their financial losses [34]. Total swap volumes, including buybacks and other exchanges, peaked in 1990 at USD 27 billion, after which they declined in the 1990s. This was partly due to structural adjustments and an improved economic performance of many indebted countries that would increase the value of their debt titles on the secondary market and thereby make them less attractive leverage instruments for environmental groups [35, 36].

Although debt-for-nature swaps were performed at much smaller scales than debt-for-equity swaps, they raised hundreds of million of dollars for environmental projects in the 1990s. It is estimated that between 1987 and 1997 USD 134 million worth of debt allowed financing of USD 126 million in nature conservation [37]. [38] estimates the volume to total USD 163 million for the period of 1987–1995 and according to OECD estimates [39], USD 1.1 billion of conservation was financed from debt titles of USD 3.6 billion between 1991 and 2003.

In the months leading up to the climate summit in Copenhagen, Indonesia named debt swaps as a source of climate finance in informal UNFCCC consultations. Still, debt-for-climate swaps were never declared as an official climate finance instrument under the UNFCCC [39]. Nevertheless, both the United States and Italy fulfilled parts of their fast-start-finance commitments [0.5 and 11%, respectively] for 2010–2012 via debt swaps, together contributing USD 82.5 million of the USD 30 billion fast-start-finance goal of the Copenhagen Accord [41].

#### Examples of debt-for-nature swap reported by literature

Debt relief linked to environmental goals or debt-for-nature swaps is not a new story: after World War II the Paris Club comprised of major creditor countries initiated large-scale debt relief programs in the form of debt-for-equity swaps, and from 1991 onwards allowed debtors to convert their public debt into local payments for social or environmental projects. Since then, debt-for-nature swaps have raised hundreds of million of dollars for the environment.

A good example of this approach is the 2015 debt swap scheme implemented by Seychelles and a club of public and private debtors which enabled the country to cancel EUR 21.6 million in exchange for domestic investments in protection of its unique marine ecosystem against a specific commitment of the Government of Seychelles to increase marine protected area from 1 to 30% of its territorial waters [42].

Another good example was the swap between Italy and the Philippines in 2012 that entailed the cancellation of the Philippine’s public debt of EUR 2.9 million in exchange for investments in environmental protection and poverty reduction [39]. The projects that were in the areas of conservation, reforestation, agriculture, and sustainable resource management placed a particular focus on the participation of local communities. By 2019, the program is estimated to have 17,000 beneficiaries, including local farmers and fishers from predominantly poor districts.

#### Features of debt swaps

**Fig. 1:**
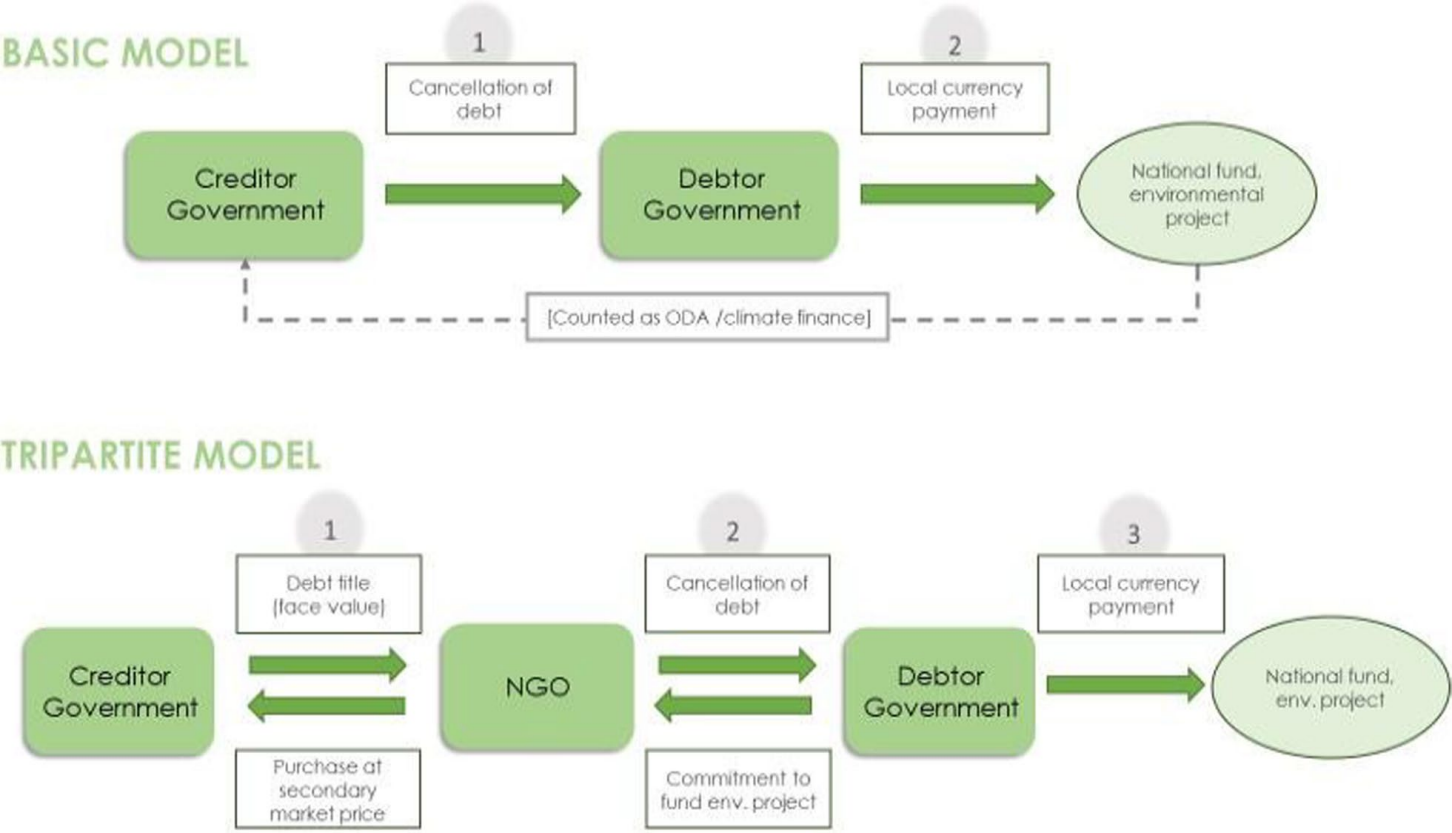
Architecture of debt-for-nature swap instruments [32]

As [32] explain, swaps are either arranged directly between one debtor and one or more creditor governments, the basic model, or are facilitated by a third party—often an international NGO tripartite model. In the latter, the NGO purchases the debt of an indebted country at a secondary market price and redeems the debt title with the debtor country in exchange for conservation efforts. The secondary market price ultimately depends on the probability of full debt repayment and is consequently higher if full repayment is expected. In addition, the extent to which the outstanding debt service payments are already written off by the creditor government as well as the overall economic situation and growth projections of the debtor governments play a role. Figure 1 provides an illustration of both models of debt-for-nature swaps.

After mutual agreement, expenditures of the debtor government are usually made gradually, often into a dedicated fund, and according to the original repayment schedule of the initial debt. They can either be channelled directly towards environmental projects or placed in a national trust fund, through which the interest earned on the deposited money can also be used to finance environmental projects, e.g., via grants to local NGOs. Such funds allow earmarking and increase accountability as they are governed by a committee composed of representatives from both governments and independent observers, such as national or international NGOs.

If debt titles are bought on the secondary market, the price is determined by the credit rating, debt situation, and overall economic performance of the indebted state. If, alternatively, debt titles are bought back via bilateral agreements, no rules or restrictions on the discount rate by which the initial debt is reduced exist. Having mainly ranged between 0 and 50% in the past, discount rates are negotiated between the participating governments on a case-by-case basis.

Overall, debt swaps are more feasible when creditor governments are willing to sell titles at a price lower than face value, because only then there is some fiscal space created for the debtor government. However, as bilateral debt is predominantly held in US dollars and investments in local environmental projects are generally made in local currency, preferable conditions could arise even at a discount rate of zero when scarce hard currency can be saved.

Most debt swaps have involved bilateral public debt, but debt swaps can also be conducted in the case of multilateral public or commercial debt. Commercial debt, for example, can be bought on the secondary market by a donor country as a form of official development assistance [ODA] or climate finance. Multilateral creditors, such as the World Bank or the IMF, cannot provide debt relief per se because of their legal status, but donor countries could use their resources to pay off the debt held at such institutions.

Debt-for-climate swaps are commonly referred to as ‘win–win’ agreements, as they benefit both debtor and creditor countries. Table 1 identifies advantages and shortfalls of debt swaps for the involved parties.

**Table 1:** Opportunities and challenges of debt swaps for the involved parties [43]

Advantages and positive outcomes for debtor country	Advantages and positive outcomes for creditor country	Shortfalls and challenges
- Through debt relief and conversion, the overall debt burden on the debtor country is lowered and the strain on the national budget is reduced - Since counterpart payments into environmental projects are generally made in local currency, debtor governments save scarce hard currency which they can then use to establish foreign exchange reserves - Debt relief can strengthen economic stability, improve the credit rating of a debtor, and attract new investments - Environmental projects benefit from freed finance that would have otherwise gone towards the creditor’s budget, often bringing economic and social benefits at a local level - Grants to environmental projects or local NGOs are typically distributed via a trust fund which is set up according to original repayment schedules. This long-term regular funding facilitates fund and, therefore, debtor’s absorption of climate finance	- From a financial perspective, creditor countries’ remaining debt claims increase in value through such swaps, and creditors can recover either full or at least part of their debt and thereby avoid the accumulation of arrears. Debt swaps are particularly beneficial if parts of the debt are already written off and full repayment is unlikely - Creditors have to mobilize less additional finance to meet their international climate commitments and, at the same time, can register the instrument as the provision of ODA. Since the nominal value of non-concessional debt can be registered as ODA, many creditor countries have used this instrument to boost their ODA numbers - Furthermore, creditor countries can raise their environmental credentials by mobilizing co-financing through international funding institutions. A debt swap that is carefully designed can guarantee an adequate use of funds and carries a greater responsibility than a single donation	- If the discount rate is low or even zero, no extra budgetary room is provided, which leaves the overall macroeconomic situation unaffected - If the debt swap volume is small, the positive impact on the debtor’s economic situation is negligible or might even be outweighed by the costs incurred when negotiating a swap and setting up a trust fund - Debtor countries must have sufficient funds to put into trust funds, and there exists a risk of inflation if debtor governments print money to pay the agreed amount in local currency - Debt swaps carry the threat of crowding out other forms of finance that are potentially more effective. Debt swaps should be additional to the already delivered ODA and not substitute other channels of new aid - Climate-relevant debt swaps have to compete with other sectors (health, education, infrastructure) for a limited amount of eligible debt

#### *Case study: Debt swap experience in Montenegro*^6^

**Fig. 2:**
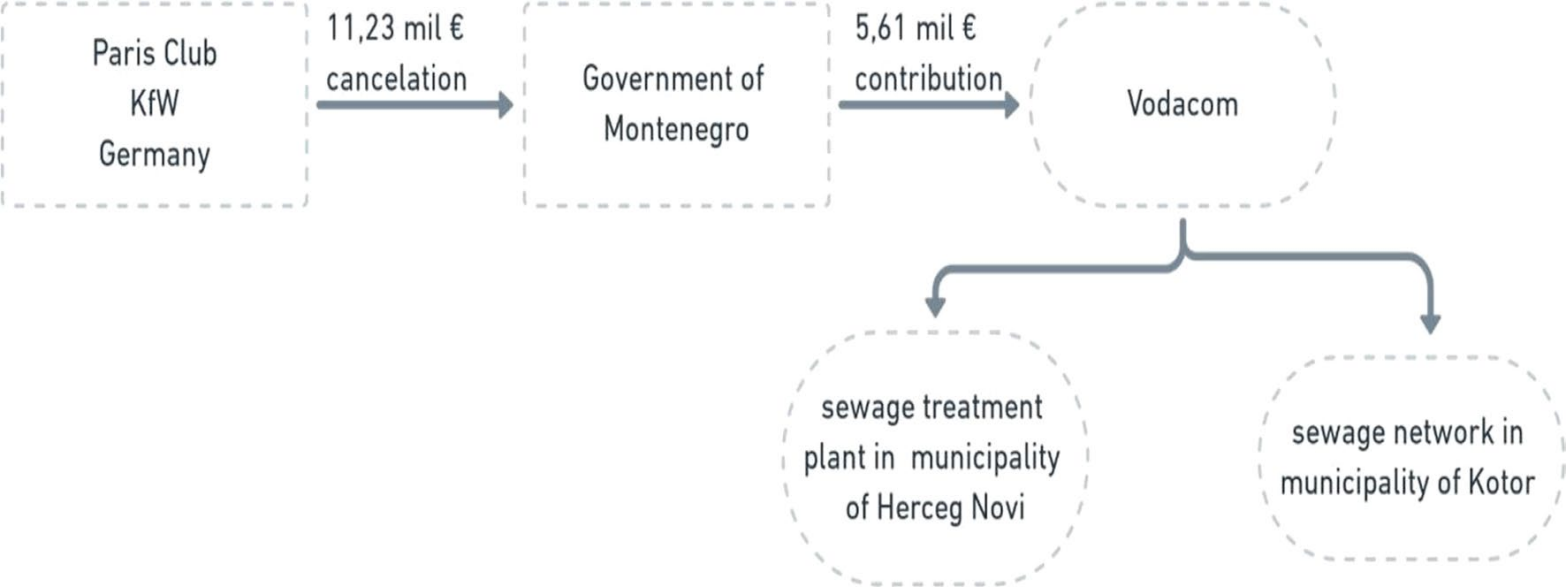
Overview of the debt to nature swap between the Paris Club and Montenegro

The Montenegrin case (Fig. 2) represents the basic model swap, since it had been arranged directly between the debtor and one creditor government. Total debt volume of EUR 11.23 million held by the government of Germany, i.e., the KfW bank as the Paris Club creditor was cancelled in exchange for a project in water supply and sanitation. The Government of Montenegro and the KfW bank negotiated the scheme of the swap and the agreement was concluded in 2008. The agreement reached by KfW bank and the Government of Montenegro envisaged cancellation of the debt amount conditioned by the fulfilment of several preconditions. The Government of Montenegro, as the debtor, was obliged to contribute an amount of EUR 5.6 million to a sewage treatment plant and sewage network in two municipalities under the Program for Water Supply and Sanitation at Adriatic Coast. Contribution was to be issued within a 1-year period from the signing of the agreement, paid into a special commercial bank account and available for the KfW inspection. Another precondition was substantial completion of a program for water supply and sanitation construction works before the end of 2012.

The final consent of the Government of Federal Republic of Germany on the KfW report on completion of program activities was the last cancellation precondition. Debtor financial obligation was transferred to a special account of the Vodacom company, organized as the Joint Service and Coordination Company for Water and Wastewater Services for the Montenegrin Coast. It was established in 2005, after the Government of Montenegro recognized the need for active work on the improvement of water supply and sewerage infrastructure on the Montenegrin Coast. The last disbursement to the provider of construction works was completed in October 2013 by Vodacom which was considered as the closure of the swap. Figure 2 illustrates the architecture of the deal.

#### Recommendations for designing debt-for-nature swaps

References [32, 43] summarize recommendations from various publications. The authors attest that environmental and fiscal improvements can only be realized when debt swaps are designed carefully, as we saw from the lessons-learned. There are three main success factors which ultimately determine the overall effectiveness of the scheme. First is the need to maximize the swap’s financial value to the debtor country to create strong political will and national buy-in. Second, the ambition of the scheme has to be aligned with the national climate goals, and a robust monitoring and reporting framework has to be put in place to ensure that its climate impacts are duly monitored and communicated. Finally, transparent governance arrangements and a well-capacitated operator of the scheme are indispensable for the success.

The first recommendation to a debtor country when designing and negotiating the financial structure of a swap mechanism to maximize the financial values of such schemes:

- Seek to achieve a positive difference between the original face value of the debt and the redemption price so that fiscal space is created. This can be done by either purchasing the debt title on the secondary market or by bilaterally agreeing on applying a discount rate greater than zero with the creditor.
- Negotiate the cancellation of the outstanding debt service payments before making counterpart payments to provide extra budgetary room.
- Convert the outstanding debt payments into local currency payments so that hard currency can be saved.
- Schedule payments according to the original repayment schedule so that a constant and predictable stream of finance is provided.
- Reinvest the interest rate earned by the funds to provide additional capitalization for the mechanism.
- Only conduct debt swaps if the debt volumes are large enough to justify the lengthy negotiation process and high transaction costs associated with deal structuring and implementation.

Furthermore, debt swaps and corresponding debt relief should be additional to the creditor’s ODA and not crowd out other ongoing investments in climate mitigation and adaptation. Second, climate-related projects, funded by debt swaps, should be additional to those already funded in debtor countries. While it is beneficial if concrete climate objectives and measures are envisioned and some infrastructure for delivering those is already established, payments originating from swap deals should not be used to legitimize cutting back governmental spending in other areas. And lastly, it is essential to ensure added finance for the debtor country through debt relief.

The second recommendation is that the design of the climate swap mechanism should be aligned with national climate commitments. In particular, they should be fully anchored in and aligned with national climate change priorities and the objectives as communicated in the National Determined Contributions [NDCs].

To ensure the achievement of climate and other environmental and social benefits of climate swap schemes, it is important to start with determining a baseline scenario against which progress and final outcomes are measured. This entails developing indicators and specific defining targets that should be set for various steps throughout the implementation phase. Monitoring plans and methodologies shall also be developed to enable regular progress tracking, reporting and communication to all involved stakeholders and the public-at-large to enhance transparency. Involvement of independent actors, such as NGOs, has proven to facilitate trust between debtor and creditor governments and has been crucial for encouraging civil society participation. While some international NGOs have gathered extensive experience in facilitating debt-for-nature swaps, the contribution of local or regional NGOs is similarly important to provide crucial insights about local conditions.

The third recommendation is that effective implementation and governance structures are essential for the success of the swap mechanism. This, first of all, calls for an establishment of a scheme operator or a selection of one from existing organizations. This should be a financial institution with solid fund management expertise and technical capacities to implement climate projects. Such a combination of financial and climate expertise rarely exists in developing countries and often has to be built from scratch with additional technical assistance from international organizations. In addition, to ensure oversight and provide strategic guidance, a good practice is to establish a supervisory committee that is composed of representatives from both the debtor government and the creditor’s as well as international and national NGOs.

The debtor government’s leading role and close involvement in designing and implementing a swap deal is crucial to ensure national ownership and longevity of the programme. At the negotiation stage, highest level political support of the climate swap proposal is particularly important for the deal to be secured. Climate-related projects should be anchored in national climate policies, and debt swaps should be embedded in a broader debt reduction strategy.

### Public debt and feasibility of instruments in Western Balkans 6

For the purpose of reviewing the public debt conditions in the Western Balkans Six [WB6] countries, the trends of debt in the 10-year period 2010–2020 was assessed.^7^ Given that the entire decade has been post-crisis, following the global financial crisis in 2008, it is to be expected that each of the countries will record an exponential trend of public debt growth. The new COVID-19 crisis period, which began in 2020 and was primarily characterized by a decline in GDP and an increase in public debt, is expected to underscore the need to finance the health sector as well as economic support programs.

What is noticeable in methodological terms is that there is no consistency in public debt reporting methodologies of the Western Balkan countries.^8^ Each country has its own approach to reporting. However, over a 10-year period, improvements in the quality and coverage of reports, as well as levels of transparency, are evident. Table 2 provides an overview of the data on public debt, its ratio to gross domestic product [GDP], as well as the structure of the public debt portfolio of the WB6 countries.

**Table 2:** Public debt portfolio of the Western Balkan 6 countries

	Montenegro			North Macedonia			Serbia			B&H			Kosovo			Albania		
	2010	2015	2020	2010	2015	2020	2010	2015	2020	2010	2015	2020	2010	2015	2020	2010	2015	2020
GDP (mil EUR)	2104	3625	4245	7109	9072	10,766	31,546	35,740	47,156	12,969	14,618	17,322	4402	5807	6831	8933	10,448	12,710
Public debt % GDP	42	61.6	102.4	24.6	38	60.2	42.9	76	56.8	33.8	40	35	6.2	13.0	24.6	57.7	72.8	77.9
Public debt portfolio (% of total public debt)																		
Multilateral and bilateral creditor loans	46.4	27.7	13.7	49.2	25	n.a	46.6	35	37.8	66.6	70.4	68.5	n.a	49.25	33	18.9	25.7	25.6
Eurobond	15.7	39.6	44.9	19	22.3	n.a	0	19	19	0	0	5.4	n.a	0	0	3.36	4.31	9.05
Private sector loans	9.8	18.1	32.5		13.6	n.a	0	5	0.9	0	4.13	1.85	n.a	0.45	1.4	3.03	6.38	3.07
Government securities	3.9	4.37	4.2	31.3	38.9	n.a	47.5	30.5	39.5	11	10.4	15.8	n.a	50.5	64.7	32.41	36.32	40.12

Source: Statistical Offices and Ministry of Finance websites of WB6 [Montenegro: www.mif.gov.me [Ministry of Finance], www.monstat.org [Statistical Office] both accessed April 2021 Serbia: www.javnidug.gov.rs [Public Debt Administration] accessed April 2021, North Macedonia: www.finance.gov.mk [Ministry of Finance], www.stat.gov.mk [Statistical Office] both accessed April 2021, Albania: www.financia.gov.al Ministry of Finance], www.instat.goval [Statistical Office] both accessed April 2021, Bosnia and Herzegovina: www.mft.gov.ba [Ministry of Finance], www.bhas.gov.me [Statistical Office] both accessed April 2021, Kosovo: www.mf.rks-gov.me [Ministry of Finance] www.ask.rks-gov.netf [Statistical Office] both accessed April 2021])

Table 2 illustrates that the condition of WB6's public debt at the end of 2020^9^ ranged widely, from 24% in Kosovo to 102% in Montenegro. The trend of the debt movement was continuously progressive, except in Serbia and Bosnia, which in the second part of the period, after 2015, managed to reduce their share of public debt in GDP. Other countries have managed to double, and some have tripled, their public debt in the past decade.

Five countries, except North Macedonia, have fiscal rules in place. All of them have a rule limiting debt, but there are differences in the size and nature of the limit. Size of the limit differs from 40% in Kosovo to 45% in Albania and Serbia to 60% in Montenegro and Bosnia and Herzegovina [44]. It is clear that all countries, except Bosnia and Herzegovina and Kosovo, have been violating this fiscal rule for some time. The COVID crisis is likely to further slow these countries' ability to comply with the public debt limit. The public debt of the WB6 countries is predominantly external, except in Kosovo, where starting in 2015, the situation is changing in favor of domestic debt. In addition, public debt is dominant and euroized in countries that have their own currency.

As for the public debt portfolio, each of the countries has certain specificities, depending on the economic and political heritage. Those who had liabilities based on old foreign currency savings, restitution and unpaid pensions have securitized them and they are present in the portfolio of their domestic debt. Each of the countries in its portfolio has loans from multilateral and bilateral creditors, government securities, euro-bonds and private sector creditors’ loans.

At the beginning of the decade, multilateral and bilateral creditors’ loans were most dominant in the public debt portfolio; and, over time, their share decreased with the exception of Bosnia and Herzegovina, where these loans represented about 70% of the total portfolio. The decrease in share of these loans in most countries was in favor of the increase in share of government securities, this time with the exception of Montenegro, which has changed the structure of its portfolio in such a way that euro-bonds and private sector loans have become the most important means of financing public debt.

Government securities involve the issuance of bonds and treasury bills. The fact that North Macedonia, Serbia, Kosovo and Albania have 30–60% of these securities in their portfolio speaks not only of domestic confidence, but also of the capacities of domestic banks and institutional investors.

The fact that all countries except Kosovo and Bosnia and Herzegovina, and some even before 2010, had successful euro-bond issues, also indicates the presence of confidence of international investors.

When it comes to the debt maturity, countries do not usually publish a single average maturity figure. Data which are mainly given refer to the maturity structure, which show that long-term^10^ securities are predominantly present in the portfolio. Specifically, Montenegro published data that the average maturity at the end of 2020 was 6.9 years, while the same data for Bosnia and Herzegovina for 2019 was 7.7 years. In addition, the ratio of debt to fixed and variable rates in most countries is in favor of fixed or is balanced.

All of the above leads to the conclusion that the Western Balkan 6 will have a specific challenge to manage their public debt in this so-called traditional way. It will be particularly interesting to see how and to what extent the governments of these countries will know how to recognize and be able to introduce new borrowing instruments.

One of the modalities recently proposed by an IMF researcher [45] is based on Klemperer’s^11^ Product-Mix Auction approach. This method holds auctions to overcome issues related to the classic sovereign debt restructuring negotiations. The application of an auction model offers a platform that enables participants to engage based on their preferences rather than a one-size-fits-all approach, which causes enormous difficulties. Such preferences may relate to bonds that are different in maturity or denominated in different currencies.

### The swap scheme’s new lease on life?

In the context of the EU accession process and the recently revealed European Green Deal, newly shaped market mechanisms for the UN2030 Agenda financing in the form of the green, social or sustainability bonds are worth considering to ascertain if new approaches are possible. This innovation may arise from an already existing debt-for-nature swap, which can trace its way back to the post WWII debt restructuring and later debt-for-equity instruments as explained in the literature review. As already shown in the case study, one of the countries that has used this opportunity was Montenegro in 2009.

On the other hand, it is evident that the EU is a willing partner and provides a different mechanism of support. The most important one, so-called Instrument of Pre-accession Assistance begins its third 2021–2027 iteration.^12^ IPA serves to provide financial assistance to the candidate countries to meet political and economic criteria for the membership and is based on the strategic documents which point out necessary reforms in the fields of the rule of law, fundamental rights and governance; socio-economic development; Union policies and acquis^13^; people-to-people contacts and reconciliation; good neighbour relations and regional cooperation. Expanding the previous IPA II mechanism to the areas of migration; security, protection of the environment and climate change should be considered as new features. In fact, the debate in Parliament during the first reading produced amendments which, assuming Paris Agreement obligations, were aimed at establishing a stronger connection between the UN 2030 Agenda and IPA. Such an approach should result in 16% of the program addressing climate impact needs of the beneficiaries. It also calls for special attention to cross-border polluting issues. Equally Parliament calls for the European Fund for Sustainable Development Plus to complement the efforts under the pre-accession programme [46, 47].^14^ As for the IPA III assistance purpose, approximately EUR 14.2 billion has been proposed and enabled along with other key areas, such as rule of law, public administration and economic governance, putting new emphasis on environment and climate action [48], which could serve as excellent leverage to generate more funding from the capital markets.

The debt analysis of the WB6 countries, despite somewhat different situations, clearly points out that there are many macro-fiscal challenges ahead and an innovative approach is needed. At the same time, despite having relatively reduced fiscal space for additional borrowing, while many structural requirements lie ahead, it is obvious that individual countries’ GDP, in purchasing power, is still significantly lagging behind the EU average, as Table 3 illustrates.

**Table 3:** WB6 GDP per capita in PPS

	% of EU average in 2019
EU 27 average	100
Montenegro	50
Serbia	41
North Macedonia	38
Bosnia and Herzegovina	32
Albania	31

Source: Eurostat https://ec.europa.eu/eurostat/web/products-datasets/

With an average score of 38^15^, in 2019, the year before the pandemic began, it is obvious how far WB6 countries need to go, and why innovative mechanisms are needed. It is only sustainable development and a continuous positive trajectory that can keep this part of the world secure and migration at bay.

However, prior to any concrete move, special attention is required to assess the legal aspects of the use of the above-described novel financial instruments—as some countries may be in differing positions. In addition, there is a need for more in-depth analysis of the particular public debt elements and their structures. This is of special relevance when it comes to the possibilities of the further use of the debt for nature swaps. It is, evidently, possible in its most intuitive form and only in case of bilateral or official development aid.

Alternatively, bringing together IPA and private markets, while combining the swap model and new forms of bond issuance, a more transparent process that require vigilant reporting and verification, the EU may further support countries of the Western Balkans by repaying part of the principal debt upon the independent verification of how proceeds are used and whether targets have been met. This approach would not be entirely new as there are already examples of the so-called direct budgetary support mechanism, which is executed against a set of policy measures a candidate country is obligated to meet.

**Fig. 3:**
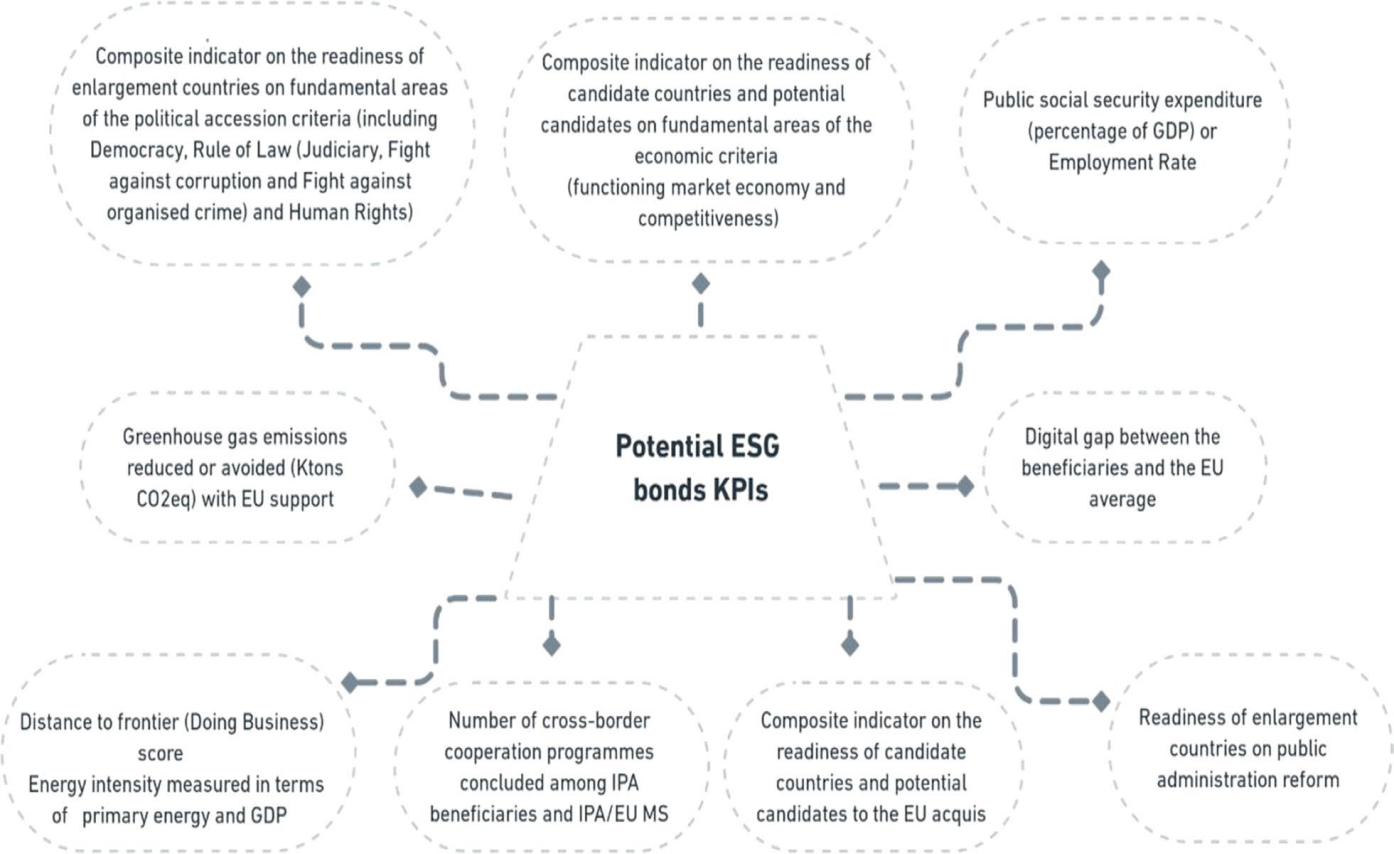
List of key performance indicators [50]

Such an approach can be directly linked with the complementary Green Agenda for Western Balkans [49], establishing a number of initiatives in different areas, such as climate change, clean energy transmission, smart and sustainable mobility, circular economy, depopulation, sustainable food systems and rural areas and the protection of biodiversity. In parallel, Western Balkan countries should continue their accession process by conducting a series of policy reviews to one day join the EU. Figure 3 presents the KPIs that are used to help measure the EU’s contribution to the achievements of the candidate countries:

It is noticeable that there is no mention of the Human Development Index produced by the UNDP, which would also be of use when tracking progress in the social sphere, related to the education and health, in particular.^16^

### Could green, social and sustainability bonds be a good fit?

**Fig. 4:**
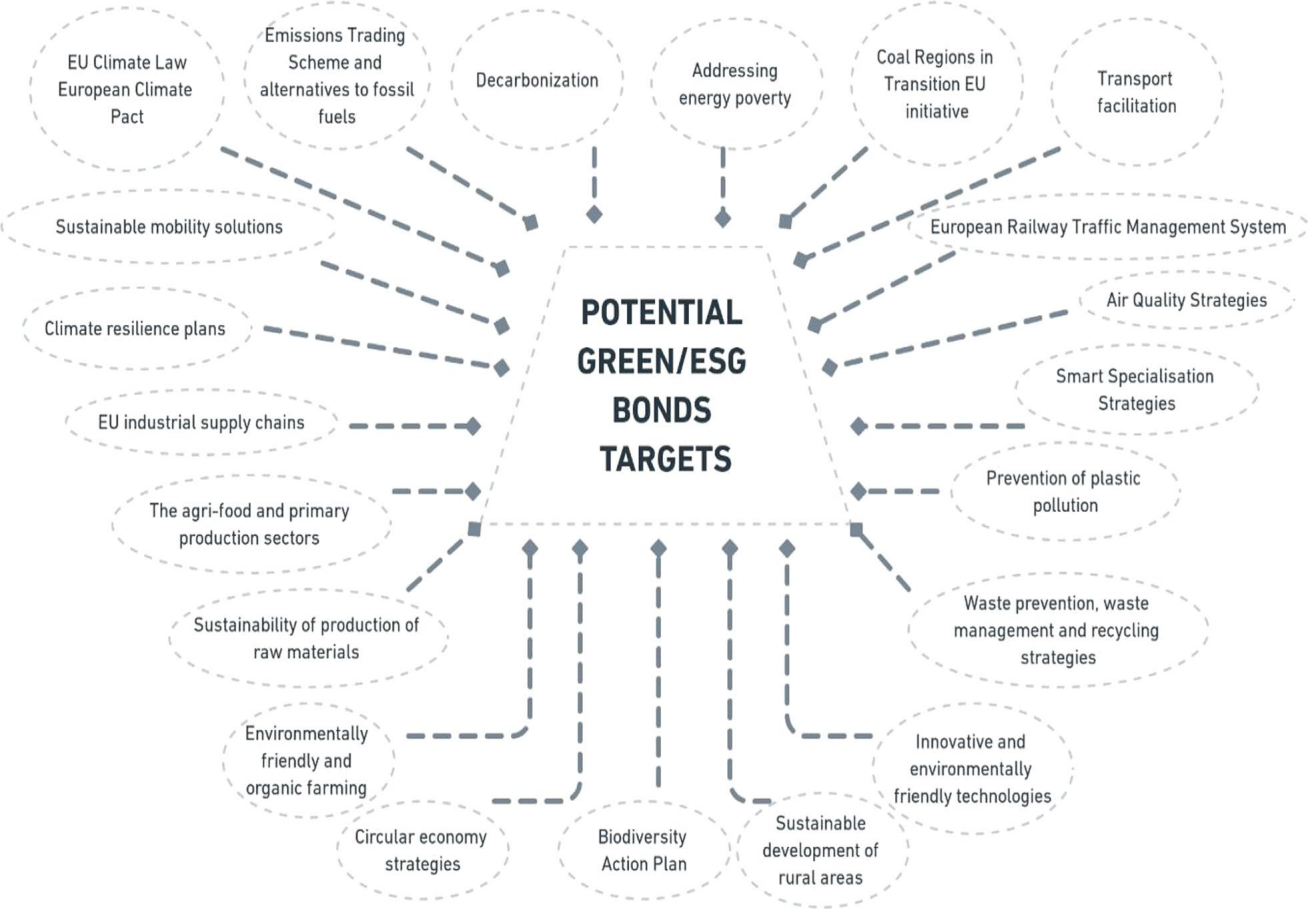
Green agenda for Western Balkans initiatives

Going back to green, social and sustainability or ESG bonds in some form could prove beneficial as they have key performance indicators and sustainable targets. Given that the European Commission uses different key performance indicators to monitor the progress countries make, those indicators could play an important role. At the same time, the Green Agenda for the Western Balkans sets off a number of initiatives that could be transferred into the targets, both indicators and these targets can serve as an excellent basis for transparency and reporting required by the markets, as it is presented in Fig. 4.

The availability of IPA III funds completes the picture as those resources can be used for the adjusted swap mechanism and to pay for the fraction of issued green, social, sustainability bonds. Paying for that in the maturity year sets free additional resources in troubled state budgets. Another option could be to issue a higher amount of bonds, while the markets realize that providing the implementation of the policies and meeting targets means that the EU would use some of the funds to repay part of the bonds once they mature. Such mechanisms would bring further relief in the lower interest that would be paid each year, which provides additional financial means to implement the EU acquis. Altogether, countries could benefit from spurring economic growth based on sustainable and smart development while keeping public debt in check.

What precise targets and what exact portion of the bonds would be repaid by the IPA III allocation would depend on the type of bond, given the varying levels of support the EU provides to different areas of investments.

Some of the initiatives may easily be turned into clear targets, some would need further work to figure out the most proper indicators. As far as the initiative related to the Biodiversity Action plan is concerned, the first target might be the adoption of a proper action plan followed by some key action points to be carried out. In addition, there has already been some research produced that could serve as useful guidance in defining a conceptual framework, such as in the example of bioenergy [51]. Besides this, there has to be a proper legislative analysis of whether issuing new bonds would require the passing of new legislation in the respective Western Balkan countries that would enable the use of the new financial instruments in the first place, similar to what Serbia experienced, prior to the new issue [21].

The work on the SDG indicators as well as the efforts and the contribution of the UN Global Compact could be of tremendous help in this space.^17^ This may also be a field that would benefit from further research.

### Summary of recommendations

**Fig. 5:**
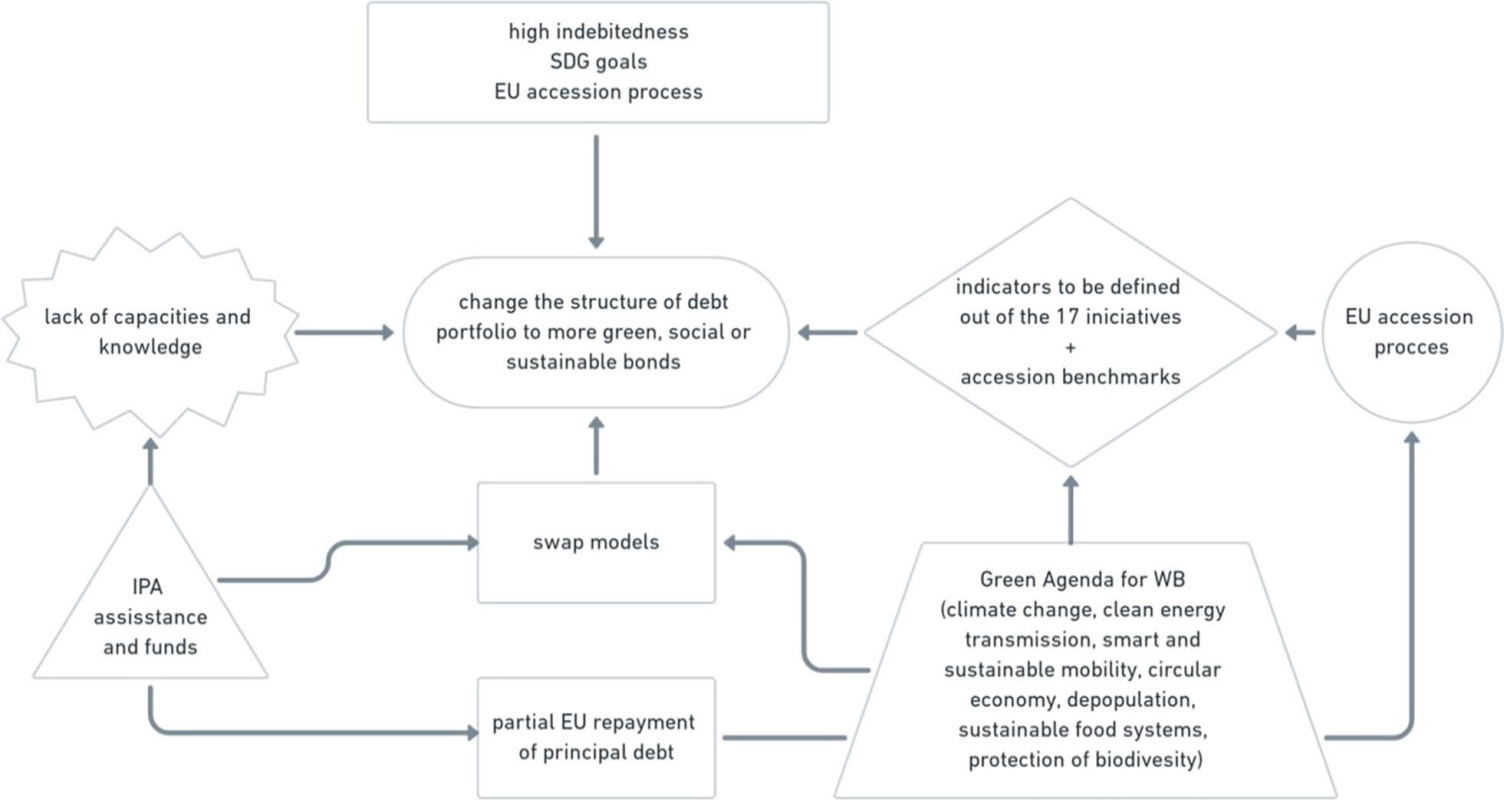
Summary of recommendations on the introduction of new financial instruments in the Western Balkans

As illustrated throughout this paper, there are plenty of different tools to use in developing innovative investment models. The option summarized below, in Fig. 5, builds on the current macroeconomic situation in the Western Balkans, explained above, which suggests that many structural issues need to be tackled by the national governments.

All the WB6 countries strive to become member states of the EU. However, that road demands the introduction of the very specific legislation which normally brings about the need to accommodate and adjust institutions, requiring additional costs and more efficient and effective public administration in the end. In addition, infrastructure and overall development needs remain very high, and macro-fiscal room has shrunk—given the levels of the public debt, in particular.

Therefore, the innovative approach presented below offers a new modality of ESG bonds which is based on the EU process combined with the Green Agenda initiatives for the Western Balkans. This new bond mechanism would use some of the lessons and practicalities learned from nature for debt swaps, where green investments could lead to some debt relief. Countries are still in the position to issue green bonds, such as the one Serbia recently released or pursue classic debt for nature swaps. The new mechanism may, in fact, serve as an extension. Countries should be encouraged to define clear targets based on the sustainable development policies and then go to the market with the transparent support of the EU through the IPA funds. The logic of the IPA funds is to prepare countries for the membership and is used to help meet various benchmarks. Even the modality by which countries benefit from the direct budget support is conditioned by some prior action.

This new approach would bring about the synergy between private and public funds; introduce very transparent targets and indicators, verifiable by independent auditors; and use IPA funds as a powerful leverage to help save some interest or principal repayment costs and thus help a country lower public debt and reinforce international financial credibility or use the extra money for additional investments in the quality of public administration or social infrastructure. Instead of chopping IPA between different sectors it would be more transparent and effective to use the contribution as an additional verification that proper policies are in place [it may combine rule of law with public administration reforms or sustainable development]. Such an approach would increase the accountability of policymakers as well.

It would be crucial to develop more measurable targets as far as the Green Agenda for the Western Balkans initiatives are concerned. Other indicators which are reported by international organizations [World Bank’s Doing Business, for example, is a more straightforward tool of assessment]. In some cases, it will require that new legislation be enacted to establish and implement these indicators. Any initiative which is measurable through additional regional mechanisms of cooperation is a value added as it contributes to the stability and prosperity of the whole region.

## Conclusion

The promise of the EU, made in 2003, that all Western Balkan countries are welcome to join the EU once established criteria are met, has set most of the policy goals for these countries. However, since then, only Croatia joined the EU on July 1, 2013. Whereas, Montenegro has advanced the most in the accession process [all the negotiating chapters are open] and Serbia following; the process overall seems to have slowed down. North Macedonia and Albania are still waiting for the real accession talks to begin, whereas Bosnia and Herzegovina and Kosovo seem to be nowhere near beginning.

In the meantime, the UN 2030 Agenda, adopted in 2015 and establishing the SDGs, and the ongoing challenges related to COVID 19 have put additional ingredients on the table. The green transition has become an equally important policy for the respective countries discussed, and along with that emerges additional social and economic challenges. Economically speaking, the situation is complex and all the Western Balkan 6 countries are faced with significant development financing needs. Their average GDP in terms of the purchase power compared to the EU average is way below 50%. While the average public debt stands at around 50%, this figure, which may seem moderate, hides the growing trend over the past decade and major discrepancies amongst countries, such as Montenegro and Albania on one hand and Kosovo, for example, on the other.

Therefore, the complex agenda involving EU accession, infrastructure and SDG needs requires innovation in the SDG financing.

This paper analyses some of the new mechanisms, such as green, social and sustainability bonds and nature for debt swaps. It is obvious that some of the main benefits of the new form of ESG bonds is a higher level of transparency and, inevitably, a higher level of accountability that comes along with it. To achieve benefits such as lower interest rates of the longer maturity terms, it is important to incorporate independent verification metrics to determine whether the various KPIs and green/social/sustainability targets are met.

On the other hand, nature for debt swaps is not entirely new and has been in place for some decades. Out of the six WB countries, it seems that only Montenegro has used it successfully and that happened once at the end of the first decade of the twenty-first century.

Putting together ESG bonds along with nature for debt swaps in light of the EU accession plans of the Western Balkans countries can offer the basis for another layer of innovative financing, and this is the key recommendation of this paper. Countries should feel encouraged to enter the private markets—clearly stating their green and de-carbonization development plans and define policy goals that stem from the European green deal and the Green agenda for the Western Balkans which has to be combined with accountability mechanisms and methodologies such as those which govern European Union accession talks with candidate countries. This recommendation assumes that participating parties will develop policy targets and indicators that are independent and verifiable, as the whole agenda grows more and more complex at each stage.

Such an innovative approach to the markets could also encourage the EU to adjust the ways pre-accession support is used [IPA III in particular] as those funds could be extremely useful leverage to access private markets and accelerate investment cycle by providing synergy between the huge opportunities private markets bring, with the crucial support of the public funds coming from the EU.

The previously discussed Paris Club debt-for-nature examples can bring another element into the picture by providing the logic of a swap, which together with a new approach to the debt sustainability offered by IMF researchers, also discussed earlier; may result in a win–win situation for all the market agents, including governments. Hopefully, this approach may unlock missing fiscal space, which in the mid- to long-term could bring about many benefits in higher levels of GDP and human development indexes.

## Data Availability

The data sets used and/or analysed during the current study are available at the following links:—Montenegro: www.mif.gov.me [Ministry of Finance], www.monstat.org [Statistical Office]—Serbia: www.javnidug.gov.rs [Public Debt Administration], -North Macedonia: www.finance.gov.mk [Ministry of Finance], www.stat.gov.mk [Statistical Office],—Albania: www.financia.gov.al [Ministry of Finance], www.instat.goval [Statistical Office],—Bosnia and Herzegovina: www.mft.gov.ba [Ministry of Finance], www.bhas.gov.me [Statistical Office],—Kosovo: www.mf.rks-gov.me [Ministry of Finance] www.ask.rks-gov.net [Statistical Office]—https://ec.europa.eu/eurostat/web/products-datasets/.

## Abbreviations

IPA: Instrument for pre-accession assistance of the EU to the candidate countries
ESG: Environmental, social and governance
KfW: Kreditanstalt fur Wiederaufbau
MDG: Millennium development goals
SDG: Sustainable development goals based on UN 2030 Agenda
UNFCCC: United Nations Framework Convention on Climate Change
UNDP: United Nations Development Program
WB6: Western Balkan 6 (Albania, Bosnia and Herzegovina, Kosovo, Montenegro, North Macedonia, Serbia)

## Footnotes

1 ESG refers to Environmental, Social and Governance, an acronym representing a growing corporate sector’s approach to the matters related to sustainability.

2 The first corporate green bond was issued in November 2013 by Vasakronan, a Swedish property company, followed by large corporate issuers include SNCF, Berlin Hyp, Apple, Engie, ICBC, and Credit Agricole. See for more information URL https://www.climatebonds.net/market/explaining-green-bonds

3 In 2018, Danone was the first corporation to issue a social bond. The proceeds (EUR 300 million) were used to fund research and development in food security. However, Emmanuel Faber, chairman of the Board who long championed social bonds and sustainable financing, was fired by the board when shareholders became unhappy with the stagnant share price, which contradicts the sustainability logic of companies working for the wider benefit to shareholders For more details, please see The Economist, Climate finance: It is not easy being green, March 27, 2021.

4 For more about the Paris Club please visit https://clubdeparis.org

5 As a consequence, then vice president of the World Wide Fund for Nature (WWF) Thomas Lovejoy proposed a debt-for-nature swap instrument that would encourage debtor countries to invest into their natural conservation programs in exchange for debt reduction [40].

6 The case study was prepared based on the inputs from the Ministry of Finance of Montenegro.

7 Is not intended, however, to execute a debt sustainability analysis as this would require separate research.

8 This is especially characteristic with the treatment of guarantees. Most countries publish public debt data without including guarantees.

9 Data for 2020 are preliminary, bearing in mind that final GDP data have not been published.

10 More than a year.

11 Paul Klemperer is a renowned economist who followed the trail of Nobel laureates Paul Milgrom and Robert Wilson and their auction analysis.

12 Agreed finally in June 2021.

13 Common term to refer to EU legislation.

14 Instrument for Pre-accession Assistance [IPA III] [46] was established in 2017 as a contribution to the pro-active development aid related to the UN2030 expected to have generated 44bn investments based on the EUR 4.1 billion initial contribution. [47]

15 The table lacks data for Kosovo, which if included would further drive the average score down.

16 www.hdr.undp.org

17 See for details https://unstats.un.org/sdgs/indicators and https://www.unglobalcompact.org
